# A Diagnostic Framework for the Management of Post-Concussion Visually Induced Dizziness: Recognising Postural and Visuoperceptual Subtypes Using an Optokinetic Balance Assessment

**DOI:** 10.3390/brainsci16070719

**Published:** 2026-07-04

**Authors:** Meyer Samuel, Beppi Carolina, Bockisch J. Christopher, Penner Marco, Straumann Dominik, Feddermann-Demont Nina

**Affiliations:** 1 Neuroscience Center Zurich, University of Zurich and ETH Zurich, 8091 Zurich, Switzerland; dominik.straumann@usz.ch; 2 Department of Neurology, University Hospital Zurich and University of Zurich, 8091 Zurich, Switzerland; carolinabeppi@gmail.com (B.C.); chris.bockisch@usz.ch (B.J.C.); marco.penner@usz.ch (P.M.); 3 Clinical Neuroscience Center, University Hospital Zurich, 8091 Zurich, Switzerland; 4 BrainCare Medical Group, 8002 Zurich, Switzerland; nina.feddermann@braincare.swiss; 5 Department of Otorhinolaryngology, Head and Neck Surgery, University Hospital Zurich and University of Zurich, 8091 Zurich, Switzerland; 6 Department of Ophthalmology, University Hospital Zurich and University of Zurich, 8091 Zurich, Switzerland; 7 Faculty of Medicine, University of Zurich, 8091 Zurich, Switzerland

**Keywords:** visually induced dizziness, visual vertigo, concussion, persisting symptoms, optokinetic stimulation, vestibular dysfunction, postural instability, clinical subtypes, vestibular rehabilitation

## Abstract

**Highlights:**

**What are the main findings?**

- The multidomain optokinetic assessment successfully objectifies visual dependence and visually induced dizziness in concussed patients, independent of traditional subjective questionnaires.
- The integration of induced postural metrics and symptom scales allows for accurate classification into distinct postural, visuoperceptual, or mixed clinical subtypes.

**What are the implications of the main findings?**

- The identification of specific VID subtypes through validated, independent assessments facilitates the design of individualised, graded rehabilitation programmes.
- Implementing this diagnostic framework into clinical practice can optimise rehabilitation outcomes and raise the standard of care for the concussed population.

**Abstract:**

Background/Objectives: Dizziness is highly prevalent in patients with concussion, with a significant proportion of these patients experiencing visually induced dizziness (VID) in complex visual environments. As current diagnoses rely primarily on subjective questionnaires, establishing objective diagnostic frameworks remains a challenge. This study aimed to quantify responses to optokinetic stimulation to explore potential clinical subtypes based on objective postural and subjective symptom measures. Methods: Concussed patients and healthy controls underwent optokinetic stimulation, with patients stratified into High and Low VVAS groups by the median Visual Vertigo Analogue Scale (VVAS) score. Induced postural metrics and symptoms were statistically compared and modelled to establish a framework for subtype classification of VID. Results: Patients with High VVAS exhibited significantly greater postural instability and higher symptom scores during optokinetic stimulation than healthy controls. While mean induced postural performance and dizziness in patients with Low VVAS were comparable to healthy controls, this patient cohort demonstrated increased heterogeneity in individual responses. Regression modelling successfully distinguished patients with High VVAS from healthy controls, achieving strong diagnostic accuracy for the postural (AUC = 0.815) and symptom-based (AUC = 0.958) models. Using this diagnostic framework, distinct postural and visuoperceptual subtypes were identified within the visual–vestibular mismatch cohort. Conclusions: This study provides a robust framework for post-concussive VID by combining objective postural metrics with subjective induced symptom scales. This classification identifies distinct clinical subtypes and characterises specific manifestations to facilitate tailored rehabilitation programmes. Future research should investigate whether these findings can guide targeted and individualised programmes to optimise rehabilitation.

## 1. Introduction

Concussions are a prevalent condition in sports. The frequency of diagnoses has substantially risen over recent decades, fostering a higher public awareness [1,2,3,4]. While many individuals recover spontaneously, persisting post-concussive symptoms lasting beyond one year are observed in more than 15% of cases and according to some estimates, this figure could be as high as 50% of patients [5,6]. The true prevalence of the condition likely represents an underestimation because the inherent heterogeneity of symptoms and findings requires the involvement of diverse specialists, which often precludes the systematic documentation of cases. Beyond this epidemiological complexity, persisting symptoms can severely compromise an individual’s quality of life and lead to daily functional disabilities. Furthermore, such complications are frequently associated with mental health challenges as well as cognitive and behavioural impairments [7,8]. As such, it is critical to develop standardised multidimensional assessment frameworks to facilitate appropriate management and the identification of relevant risk factors. By adopting precision medicine approaches that move beyond purely symptom-based evaluations, clinicians can better tailor rehabilitation strategies and reduce the substantial socioeconomic burden placed on the individual [9,10,11,12].

Common symptoms following a concussion include headache, dizziness, nausea, and fatigue. These are frequently accompanied by clinical presentations such as visual and sleep disturbances, cervical spine dysfunction, and cognitive and behavioural impairments [1]. Symptom burden is widely assumed to be a strong predictor of the overall duration of rehabilitation [13]. Visual–vestibular symptoms, including dizziness and postural unsteadiness, are observed in up to 80% of concussed patients, with chronic dizziness often associated with imbalance [9,14,15]. Furthermore, individuals with vestibular disorders as a post-concussive manifestation often report an exacerbation of symptoms in visually complex environments [16,17,18]. These triggers frequently include dynamic areas with moving elements such as supermarket aisles or traffic intersections. The Bárány Society classifies this clinical phenomenon as visually induced dizziness (VID), a condition defined by symptoms triggered by an incapacitating sensitivity to large-field, complex, or dynamic visual stimuli [19]. While identifying all subtypes of dizziness after concussion remains a primary focus of ongoing research aimed at improving treatment strategies and individuals’ health outcomes, a comprehensive and standardised assessment of VID has not yet been established, despite its clinical significance [5,9,16,20].

The development of such a method is essential to further explore the hypothesis that VID stems from maladaptive sensory reweighting processes. During recovery, this process is characterised by an overreliance on the visual system, a phenomenon commonly referred to as visual dependence [18,21,22]. However, its precise development course remains unclear. Recent research suggests that the underlying neural mechanisms likely involve altered integration within visual pathways and impaired inhibition of optokinetic information [23,24,25,26]. Under normal conditions, visual cues are integrated with proprioceptive and vestibular inputs to maintain postural control and gaze stabilisation [24,27]. Depending on the environment, these inputs are dynamically reweighted toward the most reliable system [28]. In visually complex environments, post-concussive patients may experience dizziness and instability due to a compromised central sensory integration process, which may be further exacerbated by increased cognitive demand [17,21,22,29]. Consequently, there is a critical need for further development of accurate and objective measures of visual dependence and VID to correctly identify and clinically manage such pathologies.

Assessment strategies for concussions have traditionally relied on subjective symptom reports. However, identifying and recognising the sensation of dizziness is challenging, as it depends heavily on a patient’s ability to provide an accurate self-assessment [9,10,12]. Moreover, avoidance strategies might affect the ability to accurately report such disturbances on questionnaires [30]. This highlights the necessity for a broad range of objective examinations. Accordingly, balance assessments are widely used in concussed patients to evaluate the integration of multidimensional sensory cues [9,14,18,31].

To identify visual dependence and VID beyond common questionnaires such as the Visual Vertigo Analogue Scale (VVAS), optokinetic stimulation (OKS) has been investigated as a means of quantifying postural stability and the onset of symptoms induced by a visual–vestibular mismatch (VVM) [21,29,32,33]. The findings indicate that vestibular patients exposed to OKS exhibit greater sway when facing complex moving patterns. However, several studies have either excluded concussed patients or have less frequently represented those with visually induced symptoms [17,21,29,32,34,35]. Consequently, the mean postural performance of concussed patients with VID remains unclear. This uncertainty regarding performance during optokinetic assessments limits a full understanding of this pathology, which could otherwise enhance rehabilitation strategies.

Patients with VVM disorders can be classified into different clinical subtypes [29,36]. Specifically, some individuals may primarily experience visually induced symptoms in visually demanding situations, whereas others exhibit postural instability or a combination of both. Supporting this premise, a recent update to the VVAS questionnaire covers six daily conditions across three visual categories: visual perception, active self-motion, and passive motion [37]. Similarly, patients diagnosed with persistent postural–perceptual dizziness (PPPD) were classified into visual, active-motion, and combined subtypes [38]. Beyond these classifications, the influence of covariates such as age and gender must be considered to accurately differentiate symptom presentation and postural control [39,40,41]. Accordingly, patients with chronic dizziness after a head injury may display varied performance during OKS assessments depending on their corresponding clinical subtype.

Current research indicates that the correlations between visual dependence, postural responses, and subjective symptoms often diverge. While visual dependence is frequently defined by the Rod-and-Disc test, it can also be determined through postural metrics or subjective questionnaires [21,32,34,42]. For instance, daily symptoms have shown no significant association with balance or Rod-and-Disc measures [34,35]. Conversely, induced symptoms captured by the Simulator Sickness Questionnaire (SSQ) or subjective vection intensity have been significantly associated with visual dependence measures [43,44]. Nevertheless, several studies have found that balance testing, Rod-and-Disc measures, neural activity, and nystagmus assessments are often, though not always, associated with subjective symptoms [26,35,44,45,46]. It is plausible that induced symptoms are more closely aligned with objective metrics than daily life questionnaires, which rely on a patient’s memory and self-reporting accuracy [10].

Regarding postural control, it could be argued that visual motion stimuli, such as OKS, might induce greater instability. While Lubeck et al. [42] found an increased imbalance in both static and dynamic conditions, van Ombergen et al. [29] observed increased balance responses only under the second condition, suggesting that large-field visual stimuli which can simulate self-motion are essential to elicit distinct postural responses. Interestingly, both studies reported that only moving stimulation increased the induced symptoms. These discrepancies may originate from differences in methodologies including the field of view provided by the visual stimuli, or variations in study populations. Given the potential for distinct clinical subtypes within visual dependence and VID disorders, it is essential that assessments cover both symptomatic and postural domains, even if they do not correlate for every subject. A patient may experience high levels of perceived symptoms despite maintaining relatively stable postural control, a discrepancy that likely remains dependent on individual sensitivity and stimulation intensity [44]. Despite de Vestel et al. [32] providing evidence that the VVAS questionnaires may be more effective than balance assessments in distinguishing certain chronic conditions, incorporating both subjective and objective domains is relevant for a comprehensive diagnostic profile. This integrated approach allows for high-precision and individualised clinical diagnosis, ensuring appropriate management even for possible outliers within the vestibular population [35].

Some studies have also focused on improving the accuracy of diagnostic modelling in patients with VVM. In a comprehensive assessment battery, Wright et al. [9] integrated optokinetic-induced symptoms and postural responses in a virtual environment. Their approach revealed that combining balance and symptom measures could significantly enhance diagnostic accuracy, achieving an area under the receiver operating characteristic curve (AUC-ROC) of approximately 99% in patients with mild traumatic brain injury. Evaluating standardised optokinetic assessments is essential for the development of accurate and individualised approaches and hence identifying subtypes of VVM. By combining induced symptoms with objective balance metrics, rather than relying solely on the recall of everyday symptom burden, additional critical information can be integrated into the diagnostic process. This multidomain approach represents a foundation for more targeted clinical management, enabling rehabilitation strategies to be tailored to specific clinical subtypes.

In the management of VVM and VID, current therapeutic approaches utilise various validated desensitisation strategies [47,48,49,50,51]. In principle, such interventions should follow graded and tailored exposure to visual motion to achieve optimal recovery. Accordingly, progression should rely on symptom responses to the applied therapeutic programmes [1]. Hence, it is essential to adequately assess patients with post-concussive symptoms, as potentially different clinical subtypes would require distinct management. The successful implementation of such tailored programmes depends on a reliable, validated classification of both objective postural control and subjective symptoms.

Therefore, the primary aim of this study was to compare healthy individuals with concussed patients presenting mild to severe visual–vestibular symptoms. This comparison will enable us to evaluate the impact on both postural stability and subjective symptom induction during OKS, while further investigating the correlation between these measures. Furthermore, this study sought to determine whether the assessment can identify distinct postural and visuoperceptual subtypes through a multistage modelling approach, ultimately facilitating the optimisation of future rehabilitation strategies.

## 2. Materials and Methods

### 2.1. Experimental Design and Setting

This cross-sectional study was performed on consecutive patients with post-concussive persisting symptoms [52]. The control group consisted of healthy participants recruited from the University of Zurich and the surrounding community. Patients who presented to BrainCare, a specialised centre for concussion and sports neurology, between March 2024 and November 2025 were retrospectively screened and included if they met the study criteria. This study was conducted in accordance with the Declaration of Helsinki and was approved by the Zurich Cantonal Ethics Committee (protocol no.: 2024-00956).

### 2.2. Participants

Patients (*n* = 39) were assessed and diagnosed during the initial examination using a comprehensive medical history intake and a routine neurological examination performed by a board-certified neurologist. While some patients may have participated in standard physical therapy or basic vestibular exercises prior to their specialised screening, none were receiving active physiotherapy at the time of enrolment, and none had undergone targeted optokinetic stimulation treatment. The inclusion criteria were as follows: (1) age between 18 and 40 years, (2) recent concussion, either presenting alone or in combination with whiplash-associated disorders, within 4 weeks to 18 months post-injury—with normal structural neuroimaging confirming mild injury severity—and (3) signed general consent. The exclusion criteria were as follows: (1) acute vestibular syndrome lasting at least 24 h that was clinically ruled out by the neurologist, (2) severe non-correctable visual impairment, monocular vision or manifest diplopia, (3) postural impairments unrelated to dizziness (e.g., orthopaedic, neurological, infectious diseases, other medical contexts), (4) dizziness attributed to prescribed drugs, (5) cognitive impairments compromising task comprehension, (6) a preceding history of traumatic brain injury in the last 12 months—to mitigate the confounding effects of patients’ incomplete neurological recovery—and (7) pre-existing, clinically diagnosed migraine.

Healthy controls (*n* = 23) were recruited by S.M. Their inclusion criteria were the same as the patients’ criteria number (1), (3), but not (2). The exclusion criteria for patients also applied, with all criteria assessed exclusively via self-report during pre-screening. A prior history of a concussion that resulted in persistent symptoms but had been fully resolved for more than 12 months, ensured by a full return to work or sports and an entirely symptom-free status at the time of enrolment, was not considered an exclusion criterion.

### 2.3. Optokinetic Assessment

#### 2.3.1. Protocol

All the participants completed the assessment in a single session. The Dizziness Handicap Inventory (DHI) and Hospital Anxiety and Depression Scale (HADS) were completed during the preceding clinical routine, and the Visual Vertigo Analogue Scale (VVAS) and Situational Vertigo Questionnaire (SVQ) were administered at the start of the assessment. Furthermore, baseline subjective symptom burden was collected on three 11-point scales, including dizziness, headache, and nausea [53]. The balance assessment consisted of four conditions executed in the following sequence: the baseline Romberg conditions (Eyes Open and Eyes Closed), followed by OKS rotating in both anticlockwise and clockwise directions. The Romberg conditions were performed under standard room illumination while participants faced the optokinetic disc. Each trial lasted 20 s, with an approximately 1-min break provided between conditions. Immediately following a condition, the three scales of symptom burden were reevaluated to track induced subjective changes.

#### 2.3.2. Stimulus

**Figure 1:**
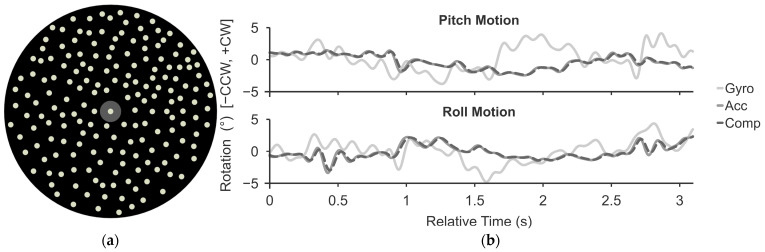
Optokinetic pattern and representative sensor signals: (**a**) The optokinetic stimulation comprises phosphorescent dots. During all conditions (Eyes Open, Eyes Closed, and OKS), participants are instructed to focus on the static dot in the grey-shaded centre. (**b**) Time-series tracking of angular orientation during the OKS. Panels display the estimated kinematics for pitch motion (top) and roll motion (bottom) for one individual measurement. Signals represent angular data from the gyroscope (Gyro), accelerometer-derived orientation estimates (Acc), and the combined output from the complementary filter (Comp). Abbreviations: OKS, optokinetic stimulation.

OKS was delivered in a completely dark environment using a custom-built 1-metre diameter physical disc (Figure 1a) [29,54]. The disc rotated at a constant speed of 30°/s and randomly covered with 179 phosphorescent dots including the dot at the centre, each approximately 1° in size [21,54,55]. The disc centre was positioned at the participants’ nose level. While standing 1.5 m from the disc, the participants focused on the centre. This setup provided a near-parallel gaze and resulted in a 36° field of view. Participants took an upright posture with their arms adducted against their sides and heels together on a firm surface, forming a 45° V-shape to provide a stable base of support [56]. To ensure participant safety during OKS, the investigator stood within arm’s reach behind the participants.

### 2.4. Outcome Measures

#### 2.4.1. Demographic Variables

Descriptive data of the healthy controls were collected prior to the optokinetic assessment. Patient data including age, gender, body mass index, time since injury and clinical diagnosis were retrieved from electronic medical records. Dizziness-related disability was assessed using the DHI total score [57,58]. The HADS questionnaire was employed to screen for anxiety (HADS-A) and depression (HADS-D), with scores of 11 or higher used to identify participants with moderate to severe symptoms [59,60].

#### 2.4.2. Clinical Visual Dependence Variables

VID in complex environments was assessed using the original Visual Vertigo Analogue Scale (VVAS), the clinical standard at study onset, and the Situational Vertigo Questionnaire (SVQ) [21,33]. The VVAS severity score served as the primary stratification factor to differentiate between patients substantially affected by VVM. To achieve this, the patient cohort was stratified into high-severity (High VVAS) and low-severity (Low VVAS) groups using a median split (median VVAS score = 8.9). Healthy controls were analysed as a distinct group, with all VVAS scores falling below a maximum of 13.5, which aligns with the observation that VVAS scores in the general population usually remain below 17 without associated functional impairment [61,62].

#### 2.4.3. Optokinetic Responses

During optokinetic balance assessment, postural stability was measured using a cost-effective inertial measurement unit consisting of an accelerometer and a gyroscope (PhidgetSpatial 3/3/3 Basic, 1042_0B, Phidget Inc., Calgary, AB, Canada), as postural responses are usually collected using high-end force plates [21,29,32,34]. The sensor was placed at the centre of the lower back at the level of the iliac crest, approximating the body’s centre of mass. Postural control data were captured at 250 Hz and saved using custom-made Python software. Moreover, symptom burden was tracked after completion of each trial.

### 2.5. Data Processing

Data processing was performed by S.M. using MATLAB (version 2024b). Raw postural sway data were processed using a fourth-order zero-phase low-pass Butterworth filter with a 10 Hz cutoff frequency [56]. A coordinate transformation was performed to align the inertial measurement unit axes with the global gravitational vector [63]. The rotation matrix was derived from the mean orientation during the first 5 s of each recording period.

A complementary filter was applied to integrate the accelerometer and gyroscope signals into a stable estimate of angular displacement using a filter coefficient of 0.8 [64]. Figure 1b demonstrates an example of the sensor fusion achieved by the complementary filter. Following sensor fusion, postural stability was quantified by the angular displacement across the pitch and roll axes, as well as the resultant radius (R) [56]. Balance metrics comprised two distinct domains of the postural response as path length was used to characterise dynamic sway and the 95% confidence ellipse area (Area_CI95_) quantified positional displacement.

Subjective symptom scores were corrected by subtracting the initial ratings from the scores recorded after each trial. To account for the overall impact of the OKS, results from clockwise and anticlockwise rotations were combined by selecting the maximum value for both postural responses and subjective symptoms.

### 2.6. Statistical Analyses

Statistical analyses were conducted using R (version 4.5.2) within the RStudio environment (version 2026.01.1). During the development and refinement of the analysis scripts, the generative artificial intelligence tool Gemini (version 3 Flash; Google LLC, Mountain View, CA, USA) was used as a technical assistant for the analysis. The generated code and final statistical outputs were manually verified and validated by the investigator to ensure computational accuracy and compliance with the study design.

Descriptive statistics are presented as means (*M*) and standard deviations (*SD*). Median values (Mdn) were calculated for variables violating the assumption of normality, as determined by the Shapiro–Wilk test. Homogeneity of variance was assessed using the Brown–Forsythe method. Univariate relationships between subjective symptom scores and objective balance metrics were examined separately for the pooled patient cohort and healthy controls using Spearman’s rank correlation coefficients (*ρ*) due to non-parametric data distributions. Statistical significance was determined after applying Benjamini–Hochberg corrections to control the False Discovery Rate across all participants and measures.

To evaluate the effects of the between-subjects factor (Group: High VVAS, Low VVAS, Healthy) and the within-subjects factor (Condition: Eyes Open, Eyes Closed, OKS), a mixed-design repeated-measures ANOVA was employed, and violations of sphericity were addressed using the Greenhouse–Geisser correction. Postural metric parameters characterised by non-normal distribution or heteroscedasticity were log_10_-transformed prior to inferential analysis (Table A1). Regarding the robustness of the *F*-test, parametric repeated-measures ANOVA was deemed appropriate for the current dataset, because the variance ratio remained below 1.7 [65]. Significant interactions were further investigated using Tukey HSD post hoc pairwise comparisons. For variables evaluated solely across the between-subjects factor, a Welch’s *t*-test or non-parametric Kruskal–Wallis test was used as appropriate to the data distribution and variance. Significant effects were followed by Dunn’s test with Benjamini–Hochberg correction for multiple comparisons.

Beyond absolute postural metrics, the Romberg (Eyes Closed to Eyes Open postural sway ratio) and kinetic quotient (OKS to Eyes Open) were also calculated and retained in the predictive modelling process. However, as they revealed no statistically significant group separation or predictive utility, they are not further reported or discussed.

A multistage regression approach was then employed to assess the diagnostic utility of the balance metrics and induced symptoms recorded during optokinetic assessment. Diagnostic models were constructed using the High VVAS group and healthy controls via Firth’s penalised likelihood logistic regression to minimise bias associated with small sample sizes [66]. All balance metrics and symptom scores were standardised using *z*-score transformation (Bessel’s correction) to ensure numerical stability and allow for the comparability of effect sizes. By calculating the transformation based on the total study cohort, the intermediate Low VVAS group was mapped onto the same numerical scale.

Following the recommendations of Luijken et al. [67], candidate parameters were selected based on a univariable inclusion threshold of *p* < 0.050. Separate multivariable models were constructed for balance metrics (Postural) and induced symptoms (Symptom) using backward elimination. These were subsequently integrated into a Combined clinical model to evaluate the synergistic accuracy of the objective and subjective markers. Demographic covariates (age, gender, body mass index) were evaluated for potential confounding. Gender and body mass index were excluded during the initial univariable screening or backward elimination process. To verify that postural metrics remained significant independent of age-related variance, a secondary analysis with strict propensity score matching (caliper = 0.8) was performed.

Model performance was quantified using the AUC-ROC and Akaike Information Criterion (AIC). Differences in diagnostic accuracy were compared using DeLong’s test for paired ROC curves. Optimal diagnostic thresholds were identified using the Youden Index (*J*), with corresponding specificity and sensitivity. Ultimately, these thresholds were used to stratify the participants based on diagnostic probability scores ranging from 0.0 to 1.0. The significance level for all tests was set at α = 0.050.

## 3. Results

### 3.1. Participants’ Characteristics

Healthy controls were aged between 22 and 40 years (*n* = 23, 13 females, *M*_age_ = 33.4 years, *SD*_age_ = 5.3 years) and reported no frequent dizziness or vertigo. Patients who presented to the outpatient neurological centre were screened for inclusion and exclusion criteria. Thirty-nine patients (20 females) with persisting post-concussive symptoms were retrospectively included in the study and completed all study assessments. Only one patient had missing body mass index data. However, this patient was included because of the small sample size. The range of diagnoses of patients with concussion is listed in Table 1. Patients frequently presented with multiple diagnoses, with all neck injuries occurring concurrently with a brain injury. Average time since injury was 161 days, ranging from 29 days to 1.5 years for all patients with persisting post-concussive symptoms. Based on the VVAS questionnaire, the patient cohort was stratified by the median severity score of 8.9, with 19 patients experiencing a range of low to severe symptoms (High VVAS: 15 females, *M*_age_ = 26.4 years, *SD*_age_ = 6.9 years) and 20 patients experiencing no to mild symptoms (Low VVAS: 5 females, *M*_age_ = 26.9 years, *SD*_age_ = 6.1 years).

Demographic and clinical characteristics, as well as subjective visual complaints, are provided in Table 2. The time since injury was not significantly different between patient cohorts (*p* = 0.988). A significant interaction was found for general dizziness symptoms, as patient groups reported significantly greater symptoms than healthy controls (both DHI scores: *p* < 0.001). In addition, the High VVAS group showed significantly greater scores than the Low VVAS group. The VVAS total score was evaluated between the full patient cohort and healthy controls, revealing a significant effect with increased scores in patients (+17.2%, *p* < 0.001). Similarly, the comparative outcome of the SVQ showed a three-way group effect, with the scores for the High VVAS group significantly higher than those of the other groups. Mental state determined by the HADS questionnaire also showed significant effects on both subscales (HADS-A: *p* = 0.008, HADS-D: *p* < 0.001), while patient cohorts did not differ in the anxiety and depression subscales (both *p* > 0.050). Compared with healthy controls, the High VVAS group demonstrated significantly elevated anxiety scores, while patients overall reported significantly higher depressive scores.

### 3.2. Optokinetic Measures

#### 3.2.1. Induced Symptoms

An overview of the induced symptoms (baseline-corrected) by the OKS is provided in Table 3. Since symptom provocation occurred predominantly during OKS and not during Eyes Open or Eyes Closed conditions, only OKS-induced changes were further analysed. These data demonstrate significantly different levels of changes across the three groups, with the most pronounced effects observed in the High VVAS group. The induced dizziness scores differed significantly between the groups (*p* < 0.001). Post hoc comparison revealed that the High VVAS group reported significantly greater scores than both patients with Low VVAS (mean difference = +1.8 units) and healthy controls (+3.0 units). Additionally, patients with Low VVAS reported greater dizziness than healthy controls (+1.2 units, *p* < 0.050). Similarly, a significant group effect was found for OKS-induced headache and nausea (*p* = 0.003 and *p* = 0.041, respectively). Headache scores were significantly higher in patients with High VVAS compared with healthy controls, with no significant differences observed between the two patient groups. For nausea, the group effect was driven by the High VVAS group, showing significantly elevated scores over healthy controls.

#### 3.2.2. Postural Responses

Four distinct balance metrics using log_10_-transformation were defined: roll, pitch, total path length, and Area_CI95_. All metrics showed significant effects for condition, while significant group effects were found for all but roll path length, presenting medium-to-large effect sizes (Table A2). Significant interactions were observed for the roll, pitch and total path lengths (*p* = 0.020, *p* = 0.010, and *p* = 0.012, respectively). However, Area_CI95_ did not reach statistical significance (*p* = 0.068).

**Figure 2:**
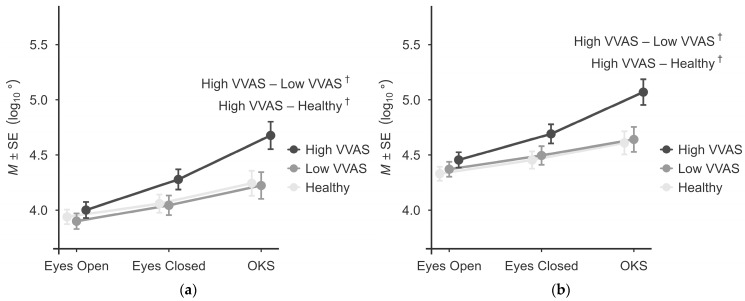
Postural responses across all groups and conditions: (**a**) Roll path length (log_10_ °). (**b**) Total path length (log_10_ °). Data for the OKS conditions represent the maximal values achieved between the clockwise and anticlockwise trials. Data points indicate the group mean, and error bars indicate the standard error. The groups are stratified based on the VVAS scores into High VVAS and Low VVAS groups. ^†^ Significantly different from High VVAS (post hoc Tukey, *p* < 0.050). Abbreviations: OKS, optokinetic stimulation; VVAS, Visual Vertigo Analogue Scale.

Post hoc comparisons revealed significant differences only in the OKS condition. In this regard, the High VVAS group exhibited significantly greater postural instability during OKS compared with healthy controls in pitch, roll, and total path length (*p* = 0.004, *p* = 0.032, and *p* = 0.013, respectively; Figure 2). Similarly, all three path lengths were increased in the High VVAS group compared with the Low VVAS group (*p* = 0.032, *p* = 0.030, and *p* = 0.027, respectively). Conversely, healthy controls and patients with Low VVAS demonstrated comparable postural responses in the OKS condition (all three metrics: *p* ≥ 0.765). Despite the lack of a significant interaction of Area_CI95_, this metric revealed the highest overall effect sizes for both the between-subject and within-subject factors.

### 3.3. Correlation

**Figure 3:**
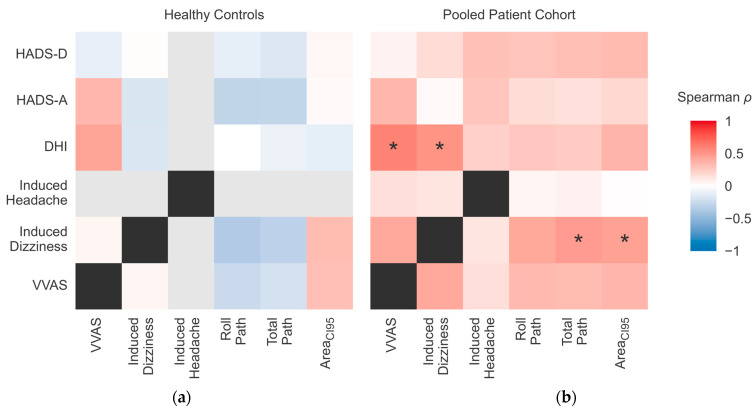
Correlation matrices for (**a**) healthy controls and (**b**) pooled patient cohort with Low and High VVAS groups. Heatmaps represent Spearman’s rank correlation coefficients (*ρ*) for healthy controls and the pooled patient cohort for selected demographic and clinical variables, as well as optokinetic measures. Red indicates a positive correlation, and blue indicates a negative correlation, as shown in the colour scale. Dark grey squares denote identical variable pairings. Area_CI95_, roll and total path length refer to log_10_-transformed postural metrics during the OKS condition. Significant correlations after Benjamini–Hochberg correction are indicated by asterisks (*p* < 0.050). Abbreviations: Area_CI95_, 95% confidence ellipse area of positional displacement; DHI, Dizziness Handicap Inventory; HADS-A, Hospital Anxiety and Depression Scale—anxiety subscale; HADS-D, Hospital Anxiety and Depression Scale—depression subscale; OKS, optokinetic stimulation; and VVAS, Visual Vertigo Analogue Scale.

Correlation analyses revealed distinct patterns of association among daily life complaints, induced symptoms, and balance metrics (Figure 3). After applying a global Benjamini–Hochberg correction across the 52 unique correlation pairs investigated, the healthy control group showed no significant associations. In the pooled patient cohort, significant relationships were observed between induced dizziness and the balance metrics, including total path length and Area_CI95_ with coefficients *ρ* ranging from 0.43 to 0.48 (all *p* < 0.048). Baseline questionnaire scores were not significantly related to postural metrics or induced symptoms in either cohort.

### 3.4. Identification of Clinical Subtypes Using Optokinetic Regression Models

To evaluate the predictive value of clinical and postural parameters for group classification (High VVAS vs. healthy controls, total *N* = 42), univariable logistic regression models using Firth’s penalised likelihood were used (Table 4). All balance metrics (roll, pitch and total path length, and Area_CI95_) emerged as significant predictors, with AUC ranging from 0.735 to 0.815. Among the induced symptoms reported during the OKS condition, induced dizziness demonstrated the highest predictive accuracy (AUC = 0.915, *p* < 0.001), whereas headache had an AUC of 0.711 (*p =* 0.039). Induced nausea showed only a trend toward significance (AUC = 0.605, *p* = 0.145). Regarding demographic covariates, age and gender were significant predictors, whereas body mass index was not (*p* = 0.894). Following this screening, parameters identified as predictors (*p* < 0.050) were selected as candidates for a subsequent multivariable regression analysis to determine the most parsimonious model for the visuoperceptual and postural domains.

#### 3.4.1. Covariates

Gender as a demographic covariate was excluded during the backward elimination process for both the Postural and Symptom models (Step 1: *p* = 0.680 and Step 1: *p* = 0.874, respectively), indicating that gender did not contribute significantly to the discriminative power of either model. Similarly, age was excluded from the Symptom model (Step 1: *p* = 0.080).

Although age was initially a significant predictor of the Postural model within the full cohort (*p* = 0.020), it was identified as a potential confounder due to baseline demographic differences between the groups. To ensure that the discriminatory power was not based on age differences, a sensitivity analysis was performed using a strictly age-matched subset (*n* = 28, Healthy: *M*_age_ = 31.1 ± 5.0 years, High VVAS: *M*_age_ = 28.5 ± 6.9 years, Welch’s *t*-test: *p* = 0.253). Within this matched cohort, the postural metric remained a significant predictor (Area_CI95_: *p* = 0.037), whereas age did not meet the criteria for inclusion in the multivariable regression analysis (Step 4: *p* = 0.641). This systematic evaluation confirmed the discriminatory power of the assessment measures and the independence of all three demographic factors.

#### 3.4.2. Logistic Regression and Diagnostic Modelling

**Figure 4:**
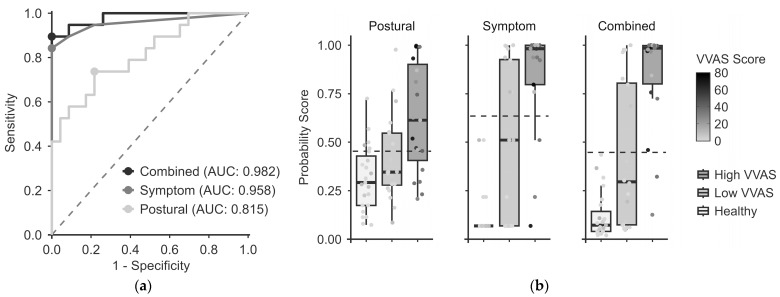
Classification performance and probability mapping of clinical subtypes: (**a**) Receiver operating characteristic curves illustrate the sensitivity and specificity of the Postural, Symptom, and Combined models, with the diagonal dashed line representing the reference line (AUC = 0.5). (**b**) Probability mapping scores for individual subjects across the three models, with colour coding representing individual VVAS scores. These distributions highlight the classification of distinct postural and visuoperceptual subtypes. Dashed horizontal lines represent the optimal diagnostic thresholds for each respective model. The groups are stratified based on the VVAS scores into High VVAS and Low VVAS groups. Abbreviations: AUC, area under the receiver operating characteristic (ROC) curve; and VVAS, Visual Vertigo Analogue Scale.

To evaluate the discriminatory power of the assessment measures regarding clinical subtypes, three logistic regression models (Postural, Symptom, and Combined) were developed using Firth’s backward elimination. The classification accuracy of each model was compared using ROC curves (Figure 4a). The Postural model identified the *z*-transformed 95% confidence ellipse area (*z*_AreaCI95_) as a unique independent predictor of clinical VVM status (*p* < 0.001). For each one-unit increase in this postural metric, the odds of being classified as having High VVAS increased by a factor of 5.94 (95% CI [1.59–22.24]). This model achieved an AUC of 0.815 (AIC = 42.5). The Symptom model yielded a substantially higher AUC of 0.958 (AIC = 24.3), with induced dizziness (*z*_Dizziness_) and headache (*z*_Headache_) identified as significant, independent predictors (*p* = 0.002 and *p* = 0.029, respectively). The presence of induced dizziness and headache increased the odds of a High VVAS classification by a factor of 14.03 (95% CI [2.66–74.09]) and 26.68 (95% CI [1.41–504.20]), respectively. Comparison via DeLong’s test revealed that the Postural and the Symptom models were not significantly different (*p* = 0.054). The Combined model, which integrated both objective postural and induced subjective predictors, demonstrated the highest overall discriminatory power (AUC = 0.982, AIC = 23.2), achieving an almost perfect separation between the two groups. The combination of the postural metric and induced symptoms resulted in a lower AIC than either model alone, supporting the utility of this multistage classification. ROC comparison between the Symptom and Combined models revealed no significant difference in accuracy (*p* = 0.468), whereas the Postural and Combined models significantly differed (*p* = 0.005). Within the Combined model, only induced dizziness remained a significant predictor (*z*_Dizziness_: *p* = 0.018), whereas induced headache and the postural metric did not (*z*_Headache_: *p* = 0.097, *z*_AreaCI95_: *p* = 0.081).

#### 3.4.3. Model Application and Probability Mapping

Optimal diagnostic thresholds, sensitivity, and specificity for these models are presented in Table 5. The Combined model achieved the highest overall performance, with a sensitivity of 0.895 and a specificity of 1.000, followed by the Symptom model (sensitivity: 0.842, specificity: 1.000) and the Postural model (sensitivity: 0.737, specificity: 0.783).

Clinical classification was performed using probability scores from the logit formulas:

- Postural model;

1.78 × *z*_AreaCI95_ ≥ −0.12 *z*_AreaCI95_: *M* ± *SD* = 0.65 ± 0.49 (1)

- Symptom model;

(2.64 × *z*_Dizziness_) + (3.28 × *z*_Headache_) ≥ −0.39
*z*_Dizziness_: *M* ± *SD* = 1.58 ± 2.00 
*z*_Headache_: *M* ± *SD* = 0.39 ± 0.88 (2)

- Combined model.

(1.30 × *z*_AreaCI95_) + (2.19 × *z*_Dizziness_) + (2.06 × *z*_Headache_) ≥ −0.74 (3)

Mapping the patient cohorts onto the diagnostic models revealed distinct patterns of classification (Figure 4b). For the High VVAS group, the Postural and Symptom models correctly identified 73.7% (*n* = 14) and 84.2% (*n* = 16) of patients, respectively. Notably, the majority of these patients (*n* = 13) were identified by both models, with only two patients (10.5%) failing to meet the classification criteria across all three models despite the initial characterisation via the VVAS questionnaire. In the Low VVAS group, the Postural and Symptom models exhibited distinct classification patterns, identifying specific clinical subtypes. While only four patients were identified by all three models, an additional eight patients were exclusively identified as sensitive within either the postural (*n* = 3) or visuoperceptual (*n* = 5) domain. The remaining eight participants (42.1%) in this cohort did not exceed the classification threshold for any model and were consistently classified as healthy. The Combined model demonstrated perfect specificity, correctly identifying 18 healthy controls. While the Postural model identified increased visual dependence in five healthy participants (21.7%), these individuals did not meet the patient criteria for either the Symptom or Combined model.

## 4. Discussion

This study presents an accurate quantification of postural stability and provocation of symptoms by OKS in patients with concussion or other head injuries, a group that is relatively underrepresented in prior research. The aim of the current study was to identify visual dependence and visually induced dizziness based on objective and subjective responses during assessments using rotating patterns. Furthermore, we hypothesised that different clinical subtypes might exist among patients with VID and VVM. Therefore, this pathology should be evaluated across multiple domains, specifically postural stability and induced symptoms, to effectively identify dysfunctional responses and add significant information to standard questionnaire findings. The results from both domains are discussed in the following sections, including the diagnostic utility of their combination in evaluating these clinical subtypes.

### 4.1. Impact on Objective Postural Stability

The findings related to postural responses are consistent with the previous literature stating that conflicting visual stimuli disturb postural stability, particularly in participants experiencing VVM and chronic dizziness [9,21,29,32,34,40,44]. Hence, patients with post-concussive symptoms, particularly those experiencing discomfort in visually demanding environments, may possess a reduced capacity to rely on vestibular and proprioceptive cues in maintaining an upright posture [22,27,28,29,32]. The neural mechanisms underlying this overreliance on vision remain a focus of current research. Increased postural instability results from maladaptive sensory reweighting, as this compensatory reaction to visual cues drives visual dependence and ultimately manifests as VID [23,24,25,26]. Potential mechanisms may involve activity-based alterations in multisensory processing networks, including the premotor, visual, vestibular, subcortical, and cerebellar areas, alongside interhemispheric pathways. Consequently, the outcomes of the current study suggest that while all participants were affected by the challenging visual stimulation, patients with High VVAS demonstrated a greater visual dependence during OKS, which was observed as increased postural instability in both the roll and pitch planes.

Significant interactions on postural control were identified across the three groups, with the High VVAS group experiencing the greatest magnitude of postural instability, while patients with Low VVAS and healthy controls performed similarly. Postural control is traditionally observed using high-end force plates, despite their limited clinical practicality and portability. The current study used an inertial measurement unit sensor, confirming its feasibility and validity to detect differences in postural responses [34,68,69]. Interestingly, while a significant main effect of condition was observed for all postural metrics, a significant interaction between group and condition was only evident for roll, pitch, and horizontal motions, whereas the positional displacement (Area_CI95_) revealed no such interaction. To ensure that post-concussive patients could complete the assessment without premature termination, a V-stance was applied providing a slightly increased area of support, thereby accounting for evidence regarding the effects of stance variation on postural control [21,56,68]. Beyond vestibular dysfunction, the complex visual demands of the OKS may have unmasked latent, compensated oculomotor deficits. While patients with manifest baseline diplopia were excluded, concussed individuals frequently exhibit convergence abnormalities or latent phorias that can decompensate under demanding visual cues, which may subsequently deteriorate postural control [70]. Although binocular vision and fixational gaze control were not explicitly tracked in the current setup, the cumulative effect on postural stability was captured, with the 95% confidence ellipse area ultimately emerging as the most effective metric for distinguishing healthy controls from patients with High VVAS within the Postural model. This finding supports the existence of a predominant postural subtype and establishes a robust baseline to help clinicians differentiate healthy responses from the dysfunctional patterns observed in patients with post-concussive symptoms.

Different clinical subtypes may exist, given that individuals often experience varying degrees of dizziness and postural instability [36]. An exacerbation can occur in several environments, such as during active self-motion in a supermarket aisle, passive motion in a car or elevator, while watching high-motion television, observing a traffic crossroads, or scrolling on mobile devices [29,38,51]. Additionally, symptoms such as brain fog and fatigue are often reported alongside dizziness, reflecting an individualised threshold for sensory overload. Indeed, a minority of healthy individuals report mild symptoms even in the absence of clinical dysfunction [16,30]. Hence, healthy controls and patients experience postural unsteadiness and visually induced symptoms to varying extents. This is further evidenced by the fact that a small minority of healthy participants exhibited positive scores on the postural domain, as defined by the established model thresholds. Consequently, the application of such comprehensive assessments using OKS is essential to capture both objective and subjective markers, thereby facilitating a more precise individual diagnosis in patients with VVM.

### 4.2. Impact on Subjective Symptom Induction

For optokinetically induced symptoms within the visuoperceptual domain, significant effects were identified for dizziness, headache, and nausea. These differences were most evident between the High VVAS and healthy control groups, with dizziness occurring almost entirely within the patient cohort, while induced headache and nausea occurred exclusively within this group. The utility of induced symptoms has been similarly investigated to identify patients with concussion and VVM, as these populations demonstrate increased symptom susceptibility during OKS [9,29,43,45]. The mechanism underlying these visuoperceptual symptoms is assumed to originate from neural maladaptive processes in patients with chronic dizziness [23,25,36]. Consequently, an overreliance on visual cues can lead to a VVM that ultimately provokes dizziness, vertigo, and postural instability. The applied assessment was specifically designed to induce such responses and replicate the complaints experienced by individuals in everyday life.

Consistent with prior research on OKS, induced symptoms offer a high potential to distinguish patients within the context of visual dependence [9,29,43,45]. In the current study, a comparison of induced symptoms revealed that the High VVAS group experienced the highest levels of subjective complaints, whereas healthy controls reported almost no dizziness or other symptoms. While dizziness was primary, patients also reported headaches and nausea. It could be argued that nausea represents a greater extent of dizziness, as described in the misery scale questionnaire, where induced dizziness and headache typically precede a nauseous sensation [29]. Moreover, the perception of symptoms may be highly individual and is usually enhanced in patient populations [29,44,61,62]. In patients with Low VVAS, a higher variability and lower predictability of induced symptoms based on questionnaire classification supported this notion. In this regard, daily complaints can be influenced by an individual’s ability to report their symptoms or the presence of avoidance strategies [10,12,30]. Collectively, the outcomes of the OKS highlight that induced symptoms are a significant marker for identifying VID, whereas headaches and nausea appear to be observable only in patients with VVM.

In general, questionnaires on daily complaints offer a simple and accessible method for rating an individual’s burden. However, these outcomes can be biased, and the association between daily complaints and symptoms reported during OKS remains inconsistent across several studies using visual motion testing [10,29,32,35]. For example, induced symptoms were only significantly related to questionnaire-based dizziness scores in concussed patients, but not in healthy controls [43]. Accordingly, the current study identified significant moderate correlations between DHI scores and both induced symptom scales and VVAS scores, but exclusively within the patient cohort. As healthy controls reported only a very low extent of daily complaints, the lack of correlation in this group was expected. Using the Rod-and-Disc test to determine visual dependence, symptoms were more frequently associated with those scores than with postural metrics, highlighting the relationship between daily complaints and induced symptoms within this context [35,40,43,45]. This trend appears to be replicated for postural stability, particularly in patients with VVM, as daily burden, induced symptoms, and postural vection ratings are positively associated with postural instability [9,29,44]. The current results support these findings regarding postural control, with a significant relationship across two out of three analysed parameters being observed only for induced symptoms in the pooled patient cohort. Healthy participants exhibited no such relationships, indicating only a few minor associations with postural metrics. Furthermore, while psychological factors are often assumed to be potential contributors to chronic dizziness, this relationship was not identified in the current study population, as shown by the absence of significant associations within the correlation matrix [21,30,35]. Nevertheless, a predominantly phobic subtype may exist within the spectrum of this disorder [36]. In summary, while a high proportion of patients may be effectively screened using standardised questionnaires, the proposed assessment provides evidence for the existence of different clinical subtypes and individual manifestations of the disorder. Consequently, it is necessary to employ assessments that capture both objective postural stability and subjective symptom induction to fully characterise individual clinical presentations.

### 4.3. Diagnostic Framework and Clinical Subtypes

To better classify the individual characterisation of this disorder, patients with post-concussive symptoms were initially categorised into two distinct cohorts using the VVAS questionnaire [33]. This approach was adopted because patients experience varying levels of symptoms, and the identification of VID in clinical practice is typically administered via a questionnaire [37,44]. Moreover, questionnaires may lack certainty due to the requirement for patients to accurately self-report and recall daily symptoms; stratification was performed using the current median VVAS severity score of 8.9 [10,62]. This was intended to build a robust model comparing healthy participants and patients with High VVAS, ultimately providing a framework to classify patients with less discriminative scores. This strategy facilitated the potential classification of clinical subtypes characterised by either postural or visuoperceptual domains, or a combination of both [29,36,37,38].

By developing diagnostic models based on OKS, this study aimed to investigate possible clinical subtypes of VID, thereby enhancing diagnostic accuracy. The modelling utility of OKS has been analysed previously in VVM and PPPD cohorts. Indeed, the clinical assessment methods and subtype classifications utilised in our framework share significant conceptual and methodological overlap with these established diagnostic criteria, despite the current focus being on the persisting post-concussive syndrome rather than a chronic, functional vestibular disorder [29,32,36]. For example, VVAS scores demonstrated the highest discriminating ability in patients with PPPD, whereas postural metrics supported the diagnosis to a lesser extent [32]. The current study excluded VVAS scores from the diagnostic models to ensure that only additional information obtained from the assessment was used to identify clinical subtypes. Including the VVAS score, which served as the basis for the initial stratification, would have introduced an inherent bias. Accordingly, previous research found that combining induced symptoms and postural responses improved the discrimination between concussed patients and healthy controls, achieving an almost perfect separation with an AUC of 0.99 [9]. Similarly, the current results revealed that the Combined model yielded the highest classification accuracy with an AUC of 0.98. In comparison, the Postural model alone achieved an AUC of 0.82, while classification based on symptoms resulted in an AUC of 0.96. By evaluating these domains separately, the assessment accounted for the existence of different clinical subtypes. There may be a predominance of either postural or visuoperceptual sensitivity, or a mixed subtype, which can eventually guide therapeutic strategies.

The current diagnostic framework across the broad range of post-concussive manifestations revealed a significant ability to account for possible clinical subtypes of VVM [1]. Both the induced postural responses and symptoms were modelled using a separate backward elimination process on the dataset of patients with High VVAS and healthy controls. It can be hypothesised that dysfunctional visual dependence resulting from an overreliance on visual cues may lead to increased instability in the postural subtype, whereas the visuoperceptual subtype predominantly experiences induced symptoms such as dizziness or vertigo [29,36,37,38]. For the postural domain, a small proportion of healthy controls showed impaired stability during OKS, indicating a possible overreliance on visual cues [62]. However, consistent with previous studies, the current findings provide evidence of a significant and substantial increase in symptom intensity observed exclusively within the patient cohorts. Accordingly, while subjective sensations are influenced by stimulation intensity and health state, patients with higher VVAS scores appear to have a lower physiological threshold in visually demanding environments [9,29,43,44]. This may explain why induced symptoms carried greater predictive weight within the diagnostic models, whereas postural responses were more susceptible to non-pathological variation in healthy individuals. However, a substantial overlap in clinical variance was observed between the subjective reports and objective balance metrics, suggesting that these clinical subtypes may not be fully separable, thereby reflecting the shared underlying mechanisms of VID.

The implementation of the Postural, Symptom, and Combined models provided a more refined classification of patients with VVM than the VVAS questionnaire alone. Specifically, the model thresholds identified 12 patients with Low VVAS scores as having either visual dependence, visually induced dizziness, or both, despite their initial classification. To illustrate the individual VVAS severity, scores were linked to the probability mapping demonstrating the questionnaire-based distribution for patients with Low VVAS (Figure 4b). This mapping revealed no clear distribution of VVAS scores across the classification in the Postural model. Similarly, a rather stochastic distribution was also found for the Symptom model, with the Combined model supporting the symptom-based outcome. This suggests that there was no clear relationship between the VVAS scores and the probability mapping across healthy controls and patients with Low VVAS. Hence, a superior diagnostic ability is achieved when using the proposed models to fully evaluate the health status of patients with VVM.

The independence of the diagnostic models was further investigated through separate analyses of several covariates. In this process, body mass index was not identified as a significant predictor for the High VVAS group. Regarding gender as another covariates, this factor is often considered for differences across tested domains in concussed patients [39]. However, that study only identified a possible trend of elevated scores in females during static balance using the Balance Error Scoring System. In the current study, gender was initially identified as a significant predictor of health status but was later eliminated during the backward modelling process for both the postural and visuoperceptual domains.

Similarly, age was detected as an initial significant predictor of the assessment outcome. However, visually induced symptoms exhibited greater predictive weight than age in the Symptom model, a finding that was not replicated for age as a factor in the Postural model. Age has been frequently investigated in balance testing, with strong evidence suggesting that it is a risk factor for visual dependence, although participants with increased instability often remain asymptomatic [41]. Therefore, explicit age matching was performed during the secondary model analysis to accurately evaluate its influence. In this process, the age factor was eliminated from the secondary Postural model during backward processing, whereas the corresponding balance metrics remained in both the full and age-matched cohort models. Accordingly, Agarwal et al. [40] reported a potential increase in visual dependence with age, but only on the Rod-and-Disc test and not within optokinetic balance testing. Consequently, the initial Postural model was constructed solely with balance metrics, highlighting the independence of these covariates in optokinetic assessment, especially within the age range investigated. The integration of both postural metrics and subjective induced measures allows for a highly sensitive classification of the two potential clinical subtypes. This offers an opportunity to add essential diagnostic information for concussed patients, ultimately providing the highest individual standards for rehabilitation.

### 4.4. Limitation

Several limitations should be considered when interpreting the current results in a broader context. Initially, optokinetic assessment and subsequent modelling of clinical subtypes were conducted on a relatively small sample size of patients with concussion or other head injuries. However, other studies in this field have used similar cohort sizes [17,21,29,35,40,43,44]. Furthermore, a patient dataset was retrospectively collected for this analysis. To mitigate this, optokinetic assessment was identically replicated in healthy controls to ensure a robust comparison in this cross-sectional study. Despite the retrospective approach, the optokinetic assessment followed a standardised, fixed-order protocol, previously applied in patients with VVM to successfully distinguish them from healthy controls, independent of side-specific effects [29]. Although randomisation of the conditions might further detail side-specific responses, this step was not the primary focus of this study.

To account for the potential bias introduced by the fixed order, only maximal measures across the optokinetic stimulation conditions were selected for group analysis. This approach aimed to isolate the greatest extent of visual-motion sensitivity, which is assumed to more closely reflect this disability in the activities of daily living. Evaluating individual participant data across all conditions revealed that while peak subjective symptoms were universally driven by OKS, the greatest postural instability occurred during a baseline Romberg condition for approximately 25% of patients and 20% of healthy controls.

In terms of gaze monitoring, oculomotor tracking was not applied to objectively record the visual fixation on the centre dot during the OKS condition. In the current clinical setup, the hardware of standard video-oculography would have obstructed the participant’s peripheral visual field and altered the stimulus delivery. Hence, participant compliance and gaze fixation were managed strictly through standardised verbal instructions. Future investigations should incorporate compatible eye-tracking technologies to quantify exact gaze metrics, nystagmus suppression, transient diplopia, or target-induced eye movements, thereby precisely evaluating their influence on postural stability [46,70].

Potential methodological bias must also be acknowledged, as the primary investigator was not blinded to the clinical diagnosis during data collection and analyses. However, the potential for bias was mitigated using automated, standardised statistical procedures and a protocol approved by the Cantonal Ethics Committee. Furthermore, all the study authors reviewed and agreed on the methodology to ensure the integrity of the findings.

Regarding the study design, it could be argued whether an assessment duration of 20 s per balance condition is sufficient, as durations of up to one minute with several replications have been recommended [56]. However, when OKS is used, an overload of induced symptoms can be provoked, potentially leading to test termination, study exclusion, or a delayed onset of severe symptoms [21,29,48]. Therefore, a conservative stimulation duration was selected for patients and was identically applied to healthy controls to avoid unacceptable symptom levels. In future studies, the assessment duration should be extended to further improve the diagnostic sensitivity of postural metrics while managing symptom induction. Nevertheless, the current findings indicate that the applied methodology is sufficiently reliable for classification purposes.

### 4.5. Clinical Relevance

The diagnostic framework developed in this study provides a standardised approach that complements subjective questionnaires with an objective assessment of postural control and induced symptoms. Identifying these two potential clinical subtypes using the proposed model offers valuable diagnostic information that supports subsequent therapeutic strategies. It is particularly important that the intensity of a consecutive desensitisation programme aligns with the assessment results, as the visuoperceptual subtype may be more susceptible to overstimulation by visual motion than the postural subtype [36,48,50]. Indeed, a rehabilitation programme should be graded and individually adapted to elicit the best possible outcome [29].

Multiple therapeutic approaches have been validated, ranging from short daily sessions to prolonged stimulation of up to one hour over six weeks [47,48,49,50,51]. As these clinical subtypes experience varying symptom levels across different visual environments, the stimulation patterns should be diversified during therapy. Notably, the current study only evaluated rotating stimuli, whereas other forms of visual motion should be considered for therapeutic applications. Specifically, symptom exacerbation during television viewing or display scrolling may not be fully addressed by a rotating pattern alone, even though such activities have been utilised as effective alternative stimulation in therapy [51]. Conversely, a postural subtype primarily characterised by an overreliance on visual inputs may benefit more from a combination of optokinetic desensitisation, balance exercises, and vestibular therapy [1]. Therefore, following a reliable assessment of VVM, it is essential to integrate these findings into appropriate and tailored rehabilitation programmes.

## 5. Conclusions

The current study demonstrates that a multidomain optokinetic assessment provides a robust framework for better objectifying visual dependence and visually induced dizziness in concussed patients, independent of the administration of standardised questionnaires. By integrating objective postural metrics with subjective induced symptoms, this methodology allows for the identification of potential visuoperceptual and postural subtypes, including mixed clinical presentations. The diagnostic models demonstrated robustness across demographic variables, including age, gender, and body mass index. Consequently, the implementation of such independent, validated assessments facilitates the design of subsequent, tailored rehabilitation programmes. By focusing treatment on distinct subtypes, clinicians can apply highly individualised, graded desensitisation and vestibular programmes, substantially improving the standards of care and recovery outcomes for the concussed population. In future research, the classification of VID subtypes should be further investigated and validated, with the potential adaptation of visual motion patterns based on these different subtypes. Moreover, specific therapeutic strategies related to diagnostic outcomes should be explored to optimise individual treatment in patients with VVM.

## Figures and Tables

**Table 1:** Clinical diagnosis distribution by symptom severity group.

Diagnoses for Head and Neck Injuries ^1^	Patients	
	High VVAS ^3^ (*n* = 19)	Low VVAS ^3^ (*n* = 20)
Brain: Concussion mTBI Commotio trunci-cerebri	20 (64.5%)	19 (52.8%)
Neck: Whiplash-associated disorder Commotio spinalis Contusio spinalis	3 (9.7%)	9 (25.0%)
Labyrinth: Commotio labyrinthi/BPPV ^2^ Contusio labyrinthi	8 (25.8%)	8 (22.2%)

^1^ Data are presented as *n* (% of the total group). Clinical diagnoses are combined within three distinct domains: brain, neck, and labyrinth. To ensure clinical accuracy, multiple diagnoses within a single domain for an individual subject are counted only once each. Because patients may present with multidomain involvement, the sum of diagnoses exceeds the total patient sample. ^2^ For the purposes of this study, patients are classified as having Commotio labyrinthi/BPPV if they develop positional vertigo after head trauma and the diagnosis is clinically verified using standard positional manoeuvres. ^3^ The groups were stratified based on the VVAS scores into High VVAS and Low VVAS groups. Abbreviations: BPPV, benign paroxysmal positional vertigo; mTBI, mild traumatic brain injury; and VVAS, Visual Vertigo Analogue Scale.

**Table 2:** Analysis of demographic and clinical variables.

Variable (Unit)	*M* ± *SD* (Mdn)			*p*-Values			
	Patients		Healthy (*n* = 23)	Group Effect	High VVAS vs. Low VVAS ^b^	High VVAS vs. Healthy ^b^	Low VVAS vs. Healthy ^b^
	High VVAS ^1^ (*n* = 19)	Low VVAS ^1^ (*n* = 20)					
Time since injury (days)	161.1 ± 150.6 (76.0)	160.4 ± 129.2 (102.5)			0.988 ^c^		
DHI score (0–100) ^a^	40.6 ± 16.7 (42.0)	18.5 ± 14.3 (15.0)	1.9 ± 4.7 (0.0)	<0.001 ***	0.007 *	<0.001 ***	<0.001 ***
VVAS score (0–100%) ^a^	38.5 ± 20.1 (34.3)	2.4 ± 2.4 (1.7)	2.8 ± 3.9 (1.1)			0.001 ***	
SVQ score (0–3) ^a^	1.67 ± 0.66 (1.79)	0.25 ± 0.23 (0.21)	0.24 ± 0.36 (0.11)	<0.001 ***	<0.001 ***	<0.001 ***	0.793
HADS-A (0–21) ^a^	6.5 ± 3.2 (6.0)	4.8 ± 2.7 (4.0)	3.7 ± 2.7 (3.0)	0.008 *	0.148	0.005 *	0.150
HADS-D (0–21) ^a^	4.9 ± 3.0 (4.0)	4.4 ± 3.6 (3.0)	1.1 ± 1.9 (0.0)	<0.001 ***	0.440	<0.001 ***	<0.001 ***

^1^ The groups are stratified based on the VVAS scores into High VVAS and Low VVAS groups. ^a^ Kruskal–Wallis test; ^b^ Dunn’s post hoc comparison; ^c^ Welch’s *t*-Test; * *p* < 0.050; *** *p* < 0.001. Abbreviations: DHI, Dizziness Handicap Inventory; HADS-A, Hospital Anxiety and Depression Scale—anxiety subscale; HADS-D, Hospital Anxiety and Depression Scale—depression subscale; Mdn, median; SVQ, Situational Vertigo Questionnaire; and VVAS, Visual Vertigo Analogue Scale.

**Table 3:** Results of induced symptoms during optokinetic stimulation.

Induced Symptoms by OKS (Range: 0–10)	*M* ± *SD* (Mdn)			*p*-Values			
	Patients		Healthy (*n* = 23)	Group Effect	High VVAS vs. Low VVAS ^b^	High VVAS vs. Healthy ^b^	Low VVAS vs. Healthy ^b^
	High VVAS ^1^ (*n* = 19)	Low VVAS ^1^ (*n* = 20)					
Dizziness ^a^	3.3 ± 1.9 (3)	1.5 ± 2.0 (0.5)	0.3 ± 0.6 (0)	<0.001 ***	0.005 *	<0.001 ***	0.050 *
Headache ^a^	0.9 ± 1.3 (0)	0.4 ± 0.6 (0)	0.0 ± 0.0 (0)	0.003 *	0.215	0.002 *	0.050
Nausea ^a^	0.9 ± 2.1 (0)	0.3 ± 1.1 (0)	0.0 ± 0.0 (0)	0.041 *	0.107	0.042 *	0.545

^1^ The groups are stratified based on the VVAS scores into High VVAS and Low VVAS groups. ^a^ Kruskal–Wallis test; ^b^ Dunn’s post hoc comparison; * *p* < 0.050; *** *p* < 0.001. Abbreviations: Mdn, median; OKS, optokinetic stimulation; and VVAS, Visual Vertigo Analogue Scale.

**Table 4:** Predictors of the univariable regression analyses.

Parameter ^1^	Estimate	SE	OR	AUC	AIC	*p*-Value
Age	−1.10	0.37	0.33 [0.16–0.69]	0.805	46.2	0.003 *
Gender ^a^	−1.49	0.68	0.23 [0.06–0.85]	0.677	53.3	0.028 *
Body mass index	0.04	0.27	1.04 [0.61–1.77]	0.512	55.4	0.894
Induced dizziness	2.69	0.78	14.79 [3.21–68.15]	0.915	29.2	<0.001 ***
Induced headache	1.80	0.87	6.07 [1.10–33.48]	0.711	45.8	0.039 *
Induced nausea	0.65	0.44	1.91 [0.80–4.55]	0.605	53.4	0.145
Roll path length (OKS) ^b^	0.72	0.36	2.06 [1.03–4.14]	0.735	51.8	0.042 *
Pitch path length (OKS) ^b^	1.03	0.42	2.79 [1.23–6.36]	0.767	48.2	0.014 *
Total path length (OKS) ^b^	0.85	0.38	2.35 [1.11–4.98]	0.755	50.3	0.026 *
Area_CI95_ (OKS) ^b^	1.78	0.67	5.94 [1.59–22.24]	0.815	42.5	0.008 *

^1^ Univariable logistic regression models (Firth’s penalised likelihood) identifying predictors for the differentiation between healthy controls and patients with High VVAS. ^a^ dummy-coded gender (female = 0, male = 1), with a negative estimate indicating a higher probability of females in the High VVAS group; ^b^ *z*-scores of log_10_-transformed postural metrics; * *p* < 0.050; *** *p* < 0.001. Abbreviations: AIC, Akaike Information Criterion; AUC, area under the receiver operating characteristic (ROC) curve; CI, confidence interval; OKS, optokinetic stimulation; OR, odds ratio; SE, standard error; and VVAS, Visual Vertigo Analogue Scale.

**Table 5:** Optimal thresholds and diagnostic performance of models identifying clinical subtypes.

Model	Threshold ^1^	Sensitivity	Specificity
Postural	0.453	0.737	0.783
Symptom	0.635	0.842	1.000
Combined	0.447	0.895	1.000

^1^ Optimal threshold is determined using the Youden Index (*J*) derived from ROC analysis of healthy participants and the High VVAS group. Abbreviations: ROC, Receiver Operating Characteristic; and VVAS, Visual Vertigo Analogue Scale.

## Data Availability

The original data presented in this study are openly available on Zenodo (https://zenodo.org/ (accessed on 10 May 2026)), https://doi.org/10.5281/zenodo.20393106.

## Abbreviations

The following abbreviations are used in this manuscript:

Area_CI95_	95% confidence ellipse area of positional displacement
AUC-ROC	Area under the receiver operating characteristic curve
AIC	Akaike Information Criterion
BPPV	Benign paroxysmal positional vertigo
DHI	Dizziness Handicap Inventory
HADS-A	Hospital Anxiety and Depression Scale—anxiety subscale
HADS-D	Hospital Anxiety and Depression Scale—depression subscale
mTBI	Mild traumatic brain injury
OKS	Optokinetic stimulation
SVQ	Situational Vertigo Questionnaire
PPPD	Persistent postural–perceptual dizziness
VID	Visually induced dizziness
VVM	Visual–vestibular mismatch
VVAS	Visual Vertigo Analogue Scale

## Appendix A

**Table A1:** Distributional characteristics and variance homogeneity of raw and log_10_-transformed postural metrics.

Parameter ^1^	Skewness ^a^		Homogeneity of Variance ^b^		Ratio of Variance ^c^	
	Raw	Log_10_-Transformed	Raw	Log_10_-Transformed	Raw	Log_10_-Transformed
Roll path length	4.13	1.64	0.002 *	0.279	1.56	1.23
Pitch path length	3.44	1.44	0.001 *	0.213	1.63	1.39
Total path length	3.86	1.62	0.001 *	0.215	1.41	1.23
Area_CI95_	7.29	1.35	<0.001 ***	0.001 *	2.04	1.66

^1^ Postural metrics are captured during the optokinetic balance assessment, evaluating both raw and log_10_-transformed distributions to assess suitability for parametric analysis. ^a^ Data distribution and asymmetry are evaluated using traditional sample skewness via the moments package (v0.14.1) in R (v4.5.2); ^b^ Homogeneity of variance is assessed using the Brown–Forsythe method (*p*-values presented); ^c^ Ratio of the largest group variance to the smallest group variance to assess variance symmetry between healthy control, Low VVAS, and High VVAS groups; * *p* < 0.050; *** *p* < 0.001. Abbreviations: VVAS, Visual Vertigo Analogue Scale.

**Table A2:** Results of postural responses during optokinetic assessment.

Postural Metric (Unit)	*M* ± *SD* (Mdn)			ANOVA Effects and Interactions ^a^			
	Patients		Healthy (*n* = 23)		*F* (*df*)	*p*	*η* ^2^ *_G_*
	High VVAS ^1^ (*n* = 19)	Low VVAS ^1^ (*n* = 20)					
Roll path length (log_10_ °)							
Eyes Open	4.00 ± 0.26 (3.96)	3.90 ± 0.28 (3.89)	3.94 ± 0.38 (3.87)	Group Condition Interaction	2.91 (2, 59) 46.94 (1.41, 83.40) 3.53 (2.83, 83.40)	0.062 <0.001 *** 0.020 *	0.071 0.153 0.026
Eyes Closed	4.28 ± 0.40 (4.28)	4.04 ± 0.33 (3.93)	4.06 ± 0.45 (4.02)				
OKS	4.68 ± 0.70 (4.44)	4.22 ± 0.47 (4.14) ^†^	4.24 ± 0.43 (4.17) ^†^				
Pitch path length (log_10_ °)							
Eyes Open	3.99 ± 0.30 (3.95)	3.93 ± 0.31 (3.88)	3.80 ± 0.32 (3.71)	Group Condition Interaction	4.17 (2, 59) 44.03 (1.52, 89.78) 3.99 (3.04, 89.78)	0.020 * <0.001 *** 0.010 *	0.103 0.124 0.025
Eyes Closed	4.19 ± 0.36 (4.15)	4.04 ± 0.32 (3.93)	3.94 ± 0.42 (3.87)				
OKS	4.55 ± 0.62 (4.44)	4.16 ± 0.42 (4.02) ^†^	4.06 ± 0.34 (4.02) ^†^				
Total path length (log_10_ °)							
Eyes Open	4.45 ± 0.27 (4.42)	4.37 ± 0.28 (4.32)	4.33 ± 0.34 (4.24)	Group Condition Interaction	3.41 (2, 59) 46.25 (1.44, 85.06) 3.91 (2.88, 85.06)	0.040 * <0.001 *** 0.012 *	0.084 0.14 0.027
Eyes Closed	4.69 ± 0.37 (4.63)	4.50 ± 0.32 (4.41)	4.45 ± 0.43 (4.43)				
OKS	5.07 ± 0.66 (4.88)	4.64 ± 0.45 (4.51) ^†^	4.61 ± 0.39 (4.55) ^†^				
Area_CI95_ (log_10_ °^2^)							
Eyes Open	1.03 ± 0.74 (1.06)	0.72 ± 0.49 (0.81)	0.13 ± 0.76 (−0.06)	Group Condition Interaction	11.94 (2, 59) 47.13 (1.72, 101.56) 2.35 (3.44, 101.56)	<0.001 *** <0.001 *** 0.068	0.226 0.182 0.022
Eyes Closed	1.51 ± 1.03 (1.33)	1.03 ± 0.48 (0.92)	0.62 ± 0.63 (0.57)				
OKS	2.32 ± 1.46 (1.84)	1.35 ± 0.81 (1.15)	0.97 ± 0.57 (0.99)				

^1^ The groups are stratified based on the VVAS scores into High VVAS and Low VVAS groups. ^a^ mixed-design repeated-measures ANOVA; * *p* < 0.050; *** *p* < 0.001; ^†^ significantly different from High VVAS (post hoc Tukey, *p* < 0.050). Abbreviations: Mdn, median; OKS, optokinetic stimulation; and VVAS, Visual Vertigo Analogue Scale.
